# Anchoring and adjusting amidst humans: Ranging behavior of Persian leopards along the Iran-Turkmenistan borderland

**DOI:** 10.1371/journal.pone.0196602

**Published:** 2018-05-02

**Authors:** Mohammad S. Farhadinia, Paul J. Johnson, David W. Macdonald, Luke T. B. Hunter

**Affiliations:** 1 Wildlife Conservation Research Unit, Department of Zoology, University of Oxford, Recanati-Kaplan Centre, Tubney House, Oxfordshire, Oxford, United Kingdom; 2 Future4Leopards Foundation, Tehran, Iran; 3 Panthera, New York, New York, United States of America; 4 School of Life Sciences, Westville Campus, University of KwaZulu-Natal, Durban, South Africa; Leibniz-Institute of Freshwater Ecology and Inland Fisheries, GERMANY

## Abstract

Understanding the space use and movement ecology of apex predators, particularly in mosaic landscapes encompassing different land-uses, is fundamental for formulating effective conservation policy. The top extant big cat in the Middle East and the Caucasus, the Persian leopard *Panthera pardus saxicolor*, has disappeared from most of its historic range. Its spatial ecology in the areas where it remains is almost unknown. Between September 2014 and May 2017, we collared and monitored six adult leopards (5 males and 1 female) using GPS-satellite Iridium transmitters in Tandoureh National Park (355 km^2^) along the Iran-Turkmenistan borderland. Using auto-correlated Kernel density estimation based on a continuous-time stochastic process for relocation data, we estimated a mean home range of 103.4 ± SE 51.8 km^2^ for resident males which is larger than has been observed in other studies of Asian leopards. Most predation events occurred in core areas, averaging 32.4 ± SE 12.7 km^2^. Although neighboring leopards showed high spatiotemporal overlap, their hunting areas were largely exclusive. Five out of six of leopards spent some time outside the national park, among human communities. Our study suggests that a national park can play an ‘anchoring’ role for individuals of an apex predator that spend some time in the surrounding human-dominated landscapes. Therefore, we envisage that instead of emphasizing either land sharing or land sparing, a combined approach can secure the viability of resilient large carnivores that are able to coexist with humans in the rugged montane landscapes of west and central Asia.

## Introduction

Wide-ranging apex predators have spatial needs that may push them to wander beyond the boundaries of protected areas [1,2]. Prey availability and environmental productivity are major factors driving predator space use [3,4]. Predator movement patterns are also regulated by their population density [1,5,6] and climatic disturbance in resource availability [7]. Ranging beyond protected areas’ boundaries is often associated with higher human-induced mortality [1,8].

The high altitude areas of west and central Asia host low densities of wild ungulates, predominantly confined to protected areas, while high number of domestic animals dominate montane pastures [9,10]. These crowded landscapes with high spatiotemporal variability in resources create formidable challenges for conservation managers attempting to reduce conflict and foster coexistence between humans and top predators.

Current conservation planning for landscapes dominated by humans has elements of two main paradigms, the “coexistence” (land sharing) versus the “separation” (land sparing) models. The “land sharing” model, in which carnivores and humans inhabit shared landscapes, is believed to have facilitated the recovery of large carnivores in Europe [11] and North America [12]. In contrast, “land sparing” through separating areas for human use from those for wildlife conservation is shown to be more efficient for a wide range of taxa and landscapes, from trees and birds [13] to the African lion *Panthera leo* [14]. Sparing such extensive tracts for large carnivores is unlikely to be possible in many parts of the world, particularly in dry areas where these animals need to range widely to access unpredictable and spatially heterogeneous resources [15].

Large cats living in the mountains of Asia, where they are threatened by habitat loss and persecution [16,17], persist at low density (usually <3 individuals/100 km^2^ [18,19]) compared to other productive landscapes [20,21]. In human-dominated montane landscapes of Asia, land sharing is essential for persistence of large felids [2]. Nonetheless, to the best of our knowledge, there is no study which has evaluated the role of established protected areas in west and central Asia’s rugged terrain, and how the current debate of land sparing and/or land sharing is relevant for the viability of large cats in a mosaic of spared/shared lands. We address this challenge with a GPS telemetry study of the Persian leopard *P*. *pardus saxicolor* in Tandoureh National Park along the Iran-Turkmenistan borderland.

Currently, only a small percentage of the extant range of leopards lies within the current network of protected areas [17]. Importantly, many Asian borderlands harbour fragmented populations of leopards [22–24] without effective transboundary conservation programs [17]. Addressing these two challenges, i.e. low protected area coverage and the lack of international cooperation is partly dependent upon a thorough understanding of the spatial and movement ecology of leopards at various political scales.

Leopards vary widely in spacing patterns across their global range influenced by densityand the predictability of resources [4,25]. Inter-individual variations, according to age, gender [1,26,27] and reproductive status [28] cause differences in ranging behaviour. Humans also can indirectly influence the spatial ecology of leopards by creating a vacuum effect whereby territories made vacant by human action cause movement from adjacent territories [29]. Decreased population density mediated by human-induced mortality can also result in inter-sexual differences in the spacing dynamics of leopards [30]. There is also a negative relationship between habitat productivity (as a proxy for prey abundance) and home range size in leopards, i.e. home-range size decreases as productivity increases [4]. Precipitation indirectly affects the ranging behavior via its influence on primary productivity and prey biomass [31].

In this paper, we used GPS data to address three objectives concerning the ranging behavior and movement ecology of Persian leopards, the top predator along the Iran-Turkmenistan borderland. We provided the first robust home range estimates for leopards in the steppe mountains of Asia. We then quantified the degree of range overlap between conspecifics of the same sex. Finally, we explored how the boundaries of a national park affected leopards’ use of space. Besides improving our understanding of leopard movement ecology and ranging behavior within a mosaic landscape of human-dominated areas and human-free national park, our findings are relevant for better management of many montane areas, where islands of small reserves are surrounded by densely populated human areas.

## Materials and methods

### Ethics statement

The study was conducted in Tandroueh National Park, Iran. The Iranian Department of Environment reviewed all sampling, trapping and handling procedures and approved permits for the work conducted (93/16270). The trapping and handling protocol was also approved by the University of Oxford’s Ethical Review Committee (BMS-ERC-160614).

### Study area

We studied leopards in Tandoureh NP, north-eastern Iran (ca. 20 km from the Turkmenistan border) from September 2014. The park has been protected since 1968 and covers 355 km^2^. It is characterized by mountains covered with wormwood *Artemisia* sp. and scattered juniper trees *Juniperus* sp. Elevation and annual precipitation range from 1,000 to 2,600 m and 250 to 300 mm, respectively.

**Fig 1:**
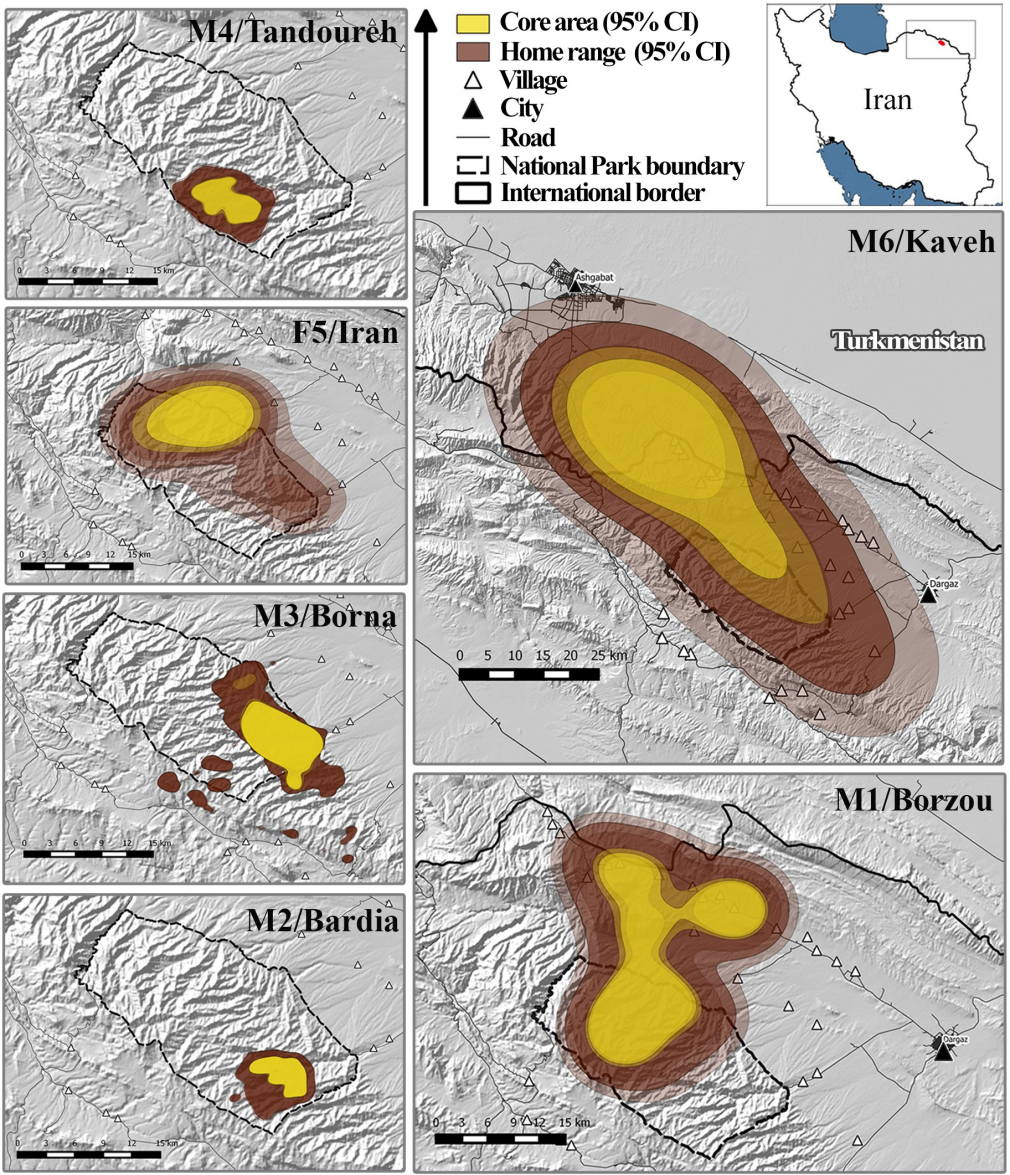
Home range of different leopard individuals tracked between September 2014 and May 2017 in Tandoureh National Park. Home ranges are delineated using auto-correlated Kernel density estimator (AKDE).

There is no human settlement inside the park. Besides the two main cities, i.e. Dargaz (Iran) and Ashgabat (the capital of Turkmenistan) near our study area (Fig 1), local communities live in villages with population ranging between 30 to 400 households. They are mainly sheep and goat herders.

The main prey species for leopards include urial *Ovis orientalis*, bezoar goat *Capra aegagrus*, and wild pig *Sus scrofa*. The availability of leopard prey in Tandoureh is affected by the national park boundaries. Wild medium-sized prey are available only inside the park, whereas domestic animals are found exclusively outside the park. The only exceptions are wild pigs, which are occasionally found in multi-use areas, outside the national park.

### Leopard capturing and collaring

We captured leopards with Aldrich foot-snares extensively modified to reduce chances of injury [32] and remotely monitored with VHF trap transmitters (Wildlife Materials, Inc., Illinois, USA) every 1–2 hours. As leopards are known to respond to baits, a wild pig carcass was used as bait, normally hanging from a tree or rock. Traps were also deployed along trails leading to the baits. In summer, we deployed traps along trails leading to water sources, sometimes without bait (see [33] for more details).

We immobilized leopards using a combination of ketamine 10% (Alfasan, Nederland BV) 2 mg/kg, medetomidine HCl 20 mg/ml (Kyron Laboratories (Pety) Ltd., Johannesburg, South Africa) 30 μg/kg and butorphanol 0.2 mg/kg (Torbugesic®, Fort Dodge Animal Health Fort Dodge Animal Health, Iowa 50501 USA) delivered intramuscularly with a dart gun (Daninject, Denmark) using a 1.5 ml dart. Trapping and handling were undertaken following approval by the University of Oxford’s Ethical Review Committee (Zool-AWERB-16062014) and the Iranian Department of Environment (93/16270).

We used GPS collars with Iridium download (LOTEK Engineering Ltd., Newmarket, ON, Canada). Each collar incorporated a drop-off buckle with a timer set to 52 weeks since deployment. Collars weighed 640 g, equivalent to less than 1–2% of leopard body mass.

Age estimates were based on dental features [34]. Anesthesia lasted for 44 to 60 minutes, followed by reversal using atipamazole (3 times the medetomidine dosage) and nantroxan (the doses equal to butorphanole), injected intramuscularly.

For programming the collars’ fix rates, we followed Knopff et al. [35] who recommended recording fixes every 3 hours to enable the identification of spatially aggregated GPS points, or clusters. However, to increase fix success rates [36] fixes were taken hourly during the last week of each month. Also, a ‘virtual fence’ option enabled us to upload the area’s boundary, so that when leopards left the defined area fix rate could be increased to hourly. Bjørneraas et al. [37] recommended that to analyze animal movement and behavior, fixes obtained immediately after collaring should be excluded because the animal is likely to behave abnormally. Therefore, we omitted the first 4 days for all collar data, associated with the earliest known kill made by the leopards after collaring (M1/Borzou).

We also investigated the potential kill sites of collared leopards. Kills were defined by clusters of GPS fixes, i.e. locations where leopards remained overnight (6 PM to 6 AM) within a radius of 200 meters. Candidate GPS clusters were investigated for possible kill remains. Prey species were categorized as “small” as < 15 kg, including red fox *Vulpes vulpes*, Indian crested porcupine *Hystrix indica* and birds or “medium” as ≥ 15 kg, such as urial, bezoar goat, wild pig, domestic sheep *Ovis aries* and domestic dog *Canis familiaris*. Young wild ungulates and domestic animals (< 1 year) were also included in medium-sized prey.

### Statistical analysis

We screened the data for two types of errors which are typical in GPS locations: missing location fixes (i.e. unsuccessful attempts of a GPS fix) and location errors of successfully acquired fixes (i.e. the difference between the recorded location and the animal's true location) [37]. After removing missing fixes, erroneous locations and outliers were screened based on identification of locations arising from unrealistic movement patterns with minimal loss of data, using a script developed by Bjørneraas et al. [37] implemented in the R environment for statistical computing [38]. We defined conservative movement values for leopards as Δ = 30,000 m; μ = 15,000 m; α = 5000 m/h; θ = −0.97 corresponding to turning angles between 166° and d 194°; Δ is a distance threshold over which an individual could not possibly travel between consecutive intervals, μ is a distance that leopard can move between two fixes and α is speed.

Multiple home range estimators are suggested to facilitate comparison with other studies that use just one method. We used three estimators for quantifying home ranges of the leopards: minimum convex polygon (MCP), kernel density estimator (KDE) and auto-correlated KDE (AKDE). Both MCP and KDE are popular for estimating animals’ home ranges, but they suffer from fundamental flaws that could degrade data quality. MCP lacks an underlying probabilistic model whereas the kernel is a nonparametric, probabilistic method, which calculates home range area based on the complete utilization distribution (UD, i.e., the probability distribution defining the animal’s use of space [39]). However, KDE assumes that the data are independent and identically distributed whereas relocation data that are ordered in time are inherently auto-correlated (i.e. an individual’s position, velocity, or acceleration measured at one point in time are statistically correlated with the same measurements in the past and future). Therefore, we also used the recently developed AKDE method, a continuous-time approach which is a fully generalized KDE to account for auto-correlated bivariate Gaussian density estimation for relocation data [40].

For each animal, we plotted an empirical variogram, which is the estimated semi-variance in positions as a function of the time lag separating observations to visually inspect the autocorrelation structure of the relocation data. Upward curvature at zero to short time lags indicates velocity autocorrelation while the long-lag behavior of the variogram illustrates space use. Thus, range residents are expected to reach an asymptote on a timescale that roughly corresponds to the home-range crossing time data [41]. In the absence of proof of range residency, we excluded them from estimating population-level movement metrics.

We used package ‘ctmm’ version 0.4.0 [41] to perform three movement models. The Independent Identically Distributed (IID) process assumes uncorrelated positions and velocities which is equal to the conventional KDE [40]. The Ornstein–Uhlenbeck (OU) process combines a random search model without space use constraint (Brownian motion) with a tendency to remain in a particular home range. Finally, the Ornstein–Uhlenbeck Foraging (OUF) process features both velocity autocorrelation time scale (a measure of path sinuosity) and restricted space use [41,42]. Both the OU and OUF model processes accommodate auto-correlated data to estimate home range size and crossing time (day).

Starting values derived from semi-variograms were used for maximum likelihood model fitting. Suitable models were fitted to the data using maximum likelihood estimation and best models were selected based on their AICc weight. The best model for each individual leopard was used to calculate movement parameters and home range; the latter defined as area within 95% % UD isopleths of AKDE estimates.

Core areas of space use, defined as the area within which an animal spends a maximum amount of time, was estimated using an individual-based quantitative approach, following Vander Wal and Rodgers [43]. Thus, the AKDE utilization distribution area was plotted against isopleths to determine the point at which the proportional home range area begins to increase at a greater rate than the probability of use (slope = 1). The value of the corresponding isopleth determines the boundary of the core area [43]. We then assessed the position of kills made by collared leopards in relation to the core area of their home ranges. We also calculated seasonal AKDE home ranges to explore variation in space use. Seasons were defined as spring = March to May, summer = June to August, fall = September to November and winter = December to February.

We used AKDE estimates to quantify an animal’s utilization distribution (UD), i.e., the probability distribution defining the animal’s use of space. Then we used AKDE’s ‘UD’ option to illustrate the ‘static interaction’, i.e. the spatial overlap of 2 home-ranges and congruence in their utilization distributions [27], ignoring the temporal sequence of movement paths [44]. Our pairwise static interaction analysis was based only on those fixes obtained from the period of time when both animals were collared, partitioned by seasons. Therefore M4 was excluded from this analysis, because he was collared after other individuals’ collars dropped off.

We calculated range overlap using function *overlap* in package ‘ctmm’ version 0.4.0 [41] which uses the Bhattacharya coefficient as an approximate measurement of the amount of overlap between two statistical samples. The overlap function incorporates movement models and calculates the overlap of their auto-correlated kernel density. For each pair of neighbors, we calculated the proportion of home range overlap of individual A on B and vice versa. A value of 1 implies that the two distributions are identical, while a value of 0 implies that the two distributions share no area in common.

In addition to home range and crossing time, two other movement parameters, the velocity autocorrelation time scale (a measure of path sinuosity) and mean distance travelled per day were also calculated [41,42] by the OUF model. All statistical analysis were implemented in R environment for statistical computing [38].

## Results

Between September 2014 and May 2017, we collared and monitored six leopards (5 males and 1 female) using GPS-satellite Iridium collars, comprising 4 adults and 2 young individuals in Tandoureh National Park. GPS collars collected between 54 and 368 days data per individual, representing a total of 56.7 monthly leopard study periods (Table 1). Our overall fix rate was high (mean 85.0% ± SE 7.6) and we obtained a total of 22226 GPS locations for 1702 leopard-days (283.7 ± SE 50.8 days/leopard). No erroneous fixes or spikes in movement were detected in our data, despite using very conservative movement parameters to screen location errors.

**Table 1:** Movement parameters and home range estimates for GPS-collared leopards in Tandoureh National Park, northeastern Iran (2014–2017). Home ranges were estimated via 95% Kernel Density Estimates (KDE) and Autocorrelated Kernel Density Estimates (AKDE). Home ranges for individuals marked with asterisk (*) were based on an Ornstein-Uhlenbeck (OU) process model, while an Ornstein-Uhlenbeck Foraging (OUF) process model was fitted to the other animals. Models were selected based on their AICc weight as calculated by ctmm package. All means for ranging metrics are calculated after removing non-resident individuals (M1, F5 and M6).

Leopard Name/ID	Sex/age	Capture date	Last day of fixes	Number of days	% days outside NP	Farthest fixes (km)	Home range crossing time (day)	Velocity autocorrelation timescale (h)	MCP 100% (km^2^)	95% KDE (km^2^)	AKDE (km^2^) (95% CI)	Core area isopleths (%)	Core area (km^2^) (95% CI)	% kills outside core area
M1/Borzou*	M/+10	5.2.2015	4.2.2016	368	43.8	30.2	4.4	NA	475.7	417.6	563.4 (448.8–690.8)	60.0	224.5 (178.8–275.3)	13.6
M2/Bardia	M/8-10	3.10.2014	30.9.2015	362	0.00	9.8	0.3	0.2	63.3	43.6	43.9 (41.2–46.7)	61.8	16.5 (15.5–17.6)	18.2
M3/Borna*	M/5-6	28.9.2014	27.9.2015	364	13.5	28.8	1.2	NA	362.2	194.2	206.5 (186.6–227.3)	67.0	57.6 (51.2–64.3)	8.7
M4/Tandoureh	M/7-10	16.8.2016	1.04.2017	228	2.20	15.3	0.6	0.1	113.9	56.8	59.8 (54.0–65.8)	61.7	23.1 (20.9–25.4)	5.3
F5/Iran	F/2-3	6.12.2015	29.1.2016	54	7.40	27.8	2.6	0.1	266.5	422.8	330.9 (208.9–480.6)	65.1	92.5 (58.4–134.3)	0.0
M6/Kaveh*	M/3-4	4.9.2015	26.8.2016	326	3.31	81.6	27.1	NA	1098.3	751.9	2269.0 (1262.4–3565.9)	62.3	775.7 (431.6–1219.0)	22.2
**Mean (SE)**				**283.7 (50.8)**	17.9 (7.3)				**179.8 (92.4)**	**98.2(48.2)**	**103.4 (51.8)**	**63.5 (1.8)**	**32.4 (12.7)**	**10.7 (3.8)**

Overall, 17.9% of GPS fixes were located outside the park (Table 1). The five collared leopards which were observed outside the park varied substantially in the amount of time spent on multi-use lands (villages, farmlands and pastures), ranging between 2.2 to 43.8% (Fig 1). Only the leopard M2/Bardia did not leave the park limits.

### Home range size and overlap

**Fig 2:**
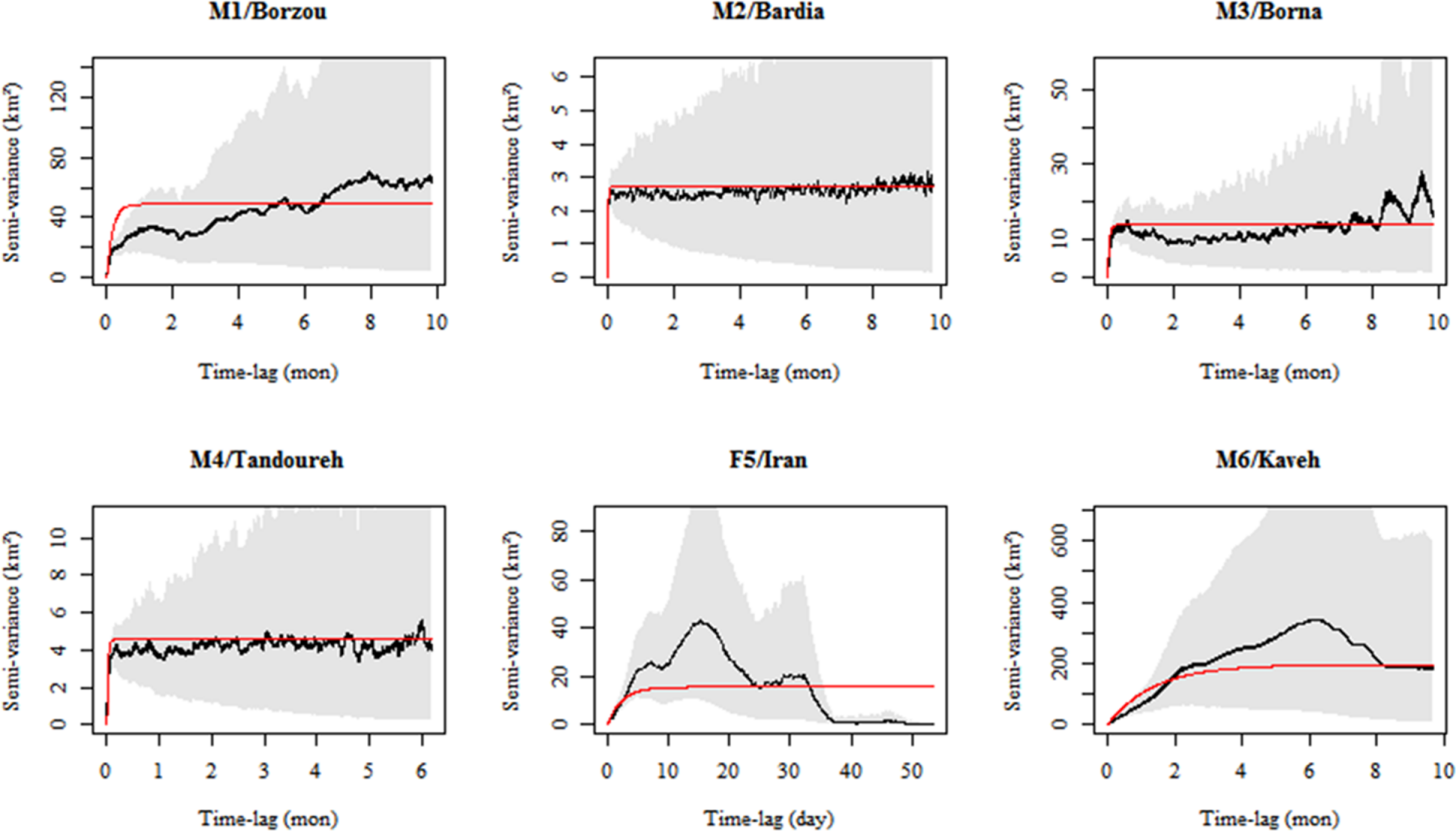
Objective assessment of residency in leopards, based on the variogram of each individual’s observed movement track. For all individuals, the fraction of the variogram displayed is 80% of the duration of each dataset, except for F5/Iran which shows the entire collaring period, i.e. 54 days.

Based on objective assessment of variograms (Fig 2), a clear asymptote was reached for three adult males M2, M3 and M4, showing their constrained space use as resident individuals. In contrast, both young leopards (F5 and M6) lacked an asymptote, evidence for lack of range residency. F5 was tracked for only 54 days which was probably not long enough to show range residency. M1 (old male) showed a mixed ranging pattern. He showed resident behavior until almost 5.5 months after collaring when his semi-variance increased and he started his excursions outside the park along the borderland’s communities with regular returns to the national park.

We excluded non-resident individuals which did not constrain their space use (F5 and M6) and the old male (M1) that appeared to become a non-resident wandering animal from the estimates of home range size. Accordingly, mean AKDE home range was calculated to be 103.4 ± SE 51.8 km^2^ for resident males which was slightly larger than their non-correlated KDE home range size estimates (1.0 to 1.1 times; Table 1). M6, possibly a dispersing young male, showed the largest range use in one year, expanding from Iran into Turkmenistan, resulting in an elongated range with 81.6 km between farthest fixes (Table 1). His AKDE analysis revealed that he finally settled in Turkmenistan, according to his core area which was placed primarily within the Turkmen territory (Fig 1). This male had the largest difference between AKDE and KDE (AKDE > 3KDE). Mean estimated core area size for resident males was 32.4 ± 12.7 km^2^, which were represented by the 62% to 67% isopleths of the utility distribution (Table 1).

**Fig 3:**
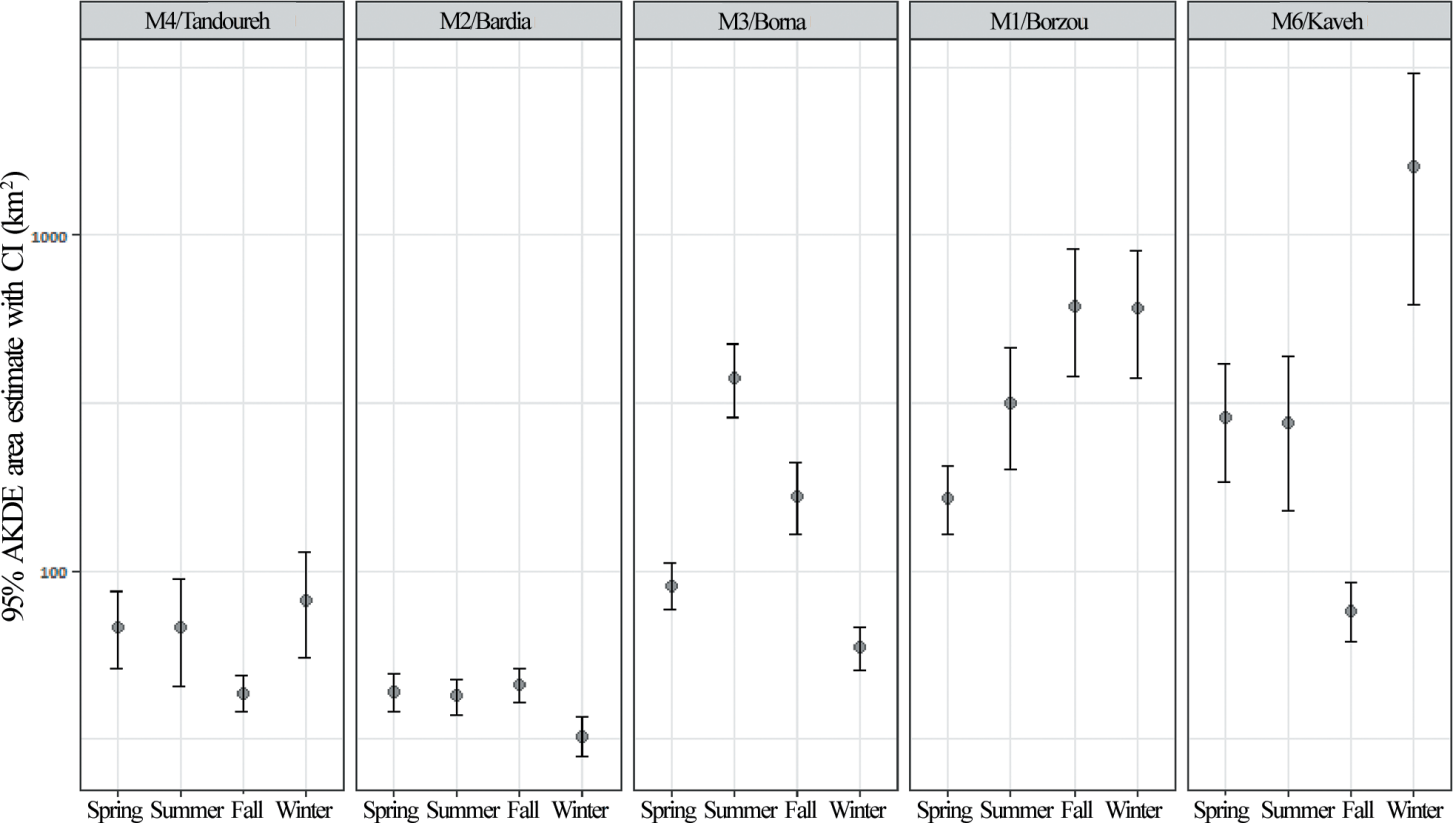
Seasonal home ranges of male Persian leopards analyzed as a continuous-time stochastic process using auto-correlated Kernel density estimator (AKDE). There is less variation in seasonal AKDE estimates for resident males (M2, M3 and M4) rather than transient individuals (M1 and M6).

There was no consistent seasonal difference in AKDE home ranges for resident males (F_5, 6_ = 1.72, *P* = 0.26). Although our sample size was small, individual variations in seasonal home range size can be seen (Fig 3). The two resident males (M2 and M3) tended to have their smallest AKDE estimates during winter when snow covered higher elevations confine their ranging to lower areas (Fig 3).

Tau (home range crossing time), varied between 0.3 and 27.1 days, and was remarkably shorter for resident (M2, M3 and M4; 0.7 SE 0.3 days) than transient leopards (M1, F5 and M6; 11.3 SE 7.9 days; Table 1). The Ornstein-Uhlenbeck Foraging (OUF) process model scored higher for three leopards based on AICc values, resulted in low directionality in movement (velocity autocorrelation time scale; Table 1).

In total, we found 139 kill remains where leopards preyed (n = 130) or scavenged (n = 9), belonging to 10 species, mostly medium sized prey (93.1%). On average, only 10.7 ± 3.8% of kills made by resident males were found outside the core areas of AKDE home ranges. No sign of predation or scavenging outside of the core area was found for the only collared female (F5/Iran) during her short tracking period while the largest proportion of outside core area’s kills belonged to the M6, the young non-resident male (22.2%, Table 1).

All leopards with neighboring ranges showed moderate home range overlap, varying from 0.29 to 0.64 (mean = 0.43 SE 0.06; Table 2). Home range overlap was also similar between resident males (0.44 SE 0.10) and resident-transient individuals (0.39 SE 0.06).

**Table 2:** Mean pairwise home range overlap estimates (confidence intervals) between neighboring leopard individuals between September 2014 and May 2017 in Tandoureh National Park, northeastern Iran. M4 was excluded from the analysis because he was collared when all other collars were dropped off.

Home range overlap	M1/Borzou(resident/transient)	M2/Bardia (resident)	M3/Borna (resident)	F5/Iran (transient)	M6/Kaveh (transient)
**M1/Borzou (resident/transient)**			0.64 (0.61–0.67)	0.49 (0.47–0.51)	0.29 (0.28–0.30)
**M2/Bardia (resident)**			0.40 (0.38–0.42)		
**M3/Borna (resident)**	0.64 (0.61–0.67)	0.40 (0.38–0.42)			
**F5/Iran (transient)**	0.49 (0.47–0.51)				0.35 (0.33–0.36)
**M6/Kaveh (transient)**	0.29 (0.28–0.30)			0.35 (0.33–0.36)	

## Discussion

Our home range estimates for Persian leopards were larger than those reported in previous Asian leopard ranging studies. Range overlap between conspecifics was relatively high and the majority of predation events occurred within home range core areas. Excursions to areas occupied by people occurred on 17.9% of all leopard collaring days with very wide variation among individuals, pointing to the importance of a combining land sparing and land sharing approaches for leopard conservation.

### Home range size

Although our data showed remarkable individual variation, leopards in Tandoureh occupied the largest home ranges recorded so far for Asian landscapes [45–47], with the exception of an adult male tracked in an arid montane habitat in central Iran (670 km^2^ [48]). The home ranges of predators scale with body mass [49] and habitat productivity, which affects prey biomass [4]. The large body masses of Persian leopards [50] and the low primary productivity of the landscapes (e.g. annual rainfall 250–300 mm in northeastern Iran) are likely to be two key determinants of their larger home range sizes.

Seasonality may partly explain variation in leopard home-range sizes at the population scale [4]. We found no evidence of consistent seasonal variation in home range size, in accordance with previous leopard studies [30,31,46,47]. Nonetheless, our data is consistent with previous observations [31,46] in suggesting that seasonal variation in home range size is an individual behavior rather than a population level trait. Both adult males (M2 and M3) restricted their ranging to lower elevations during winter when higher elevations are covered with snow and are extremely cold, both being factors known to be constraints for leopard habitat selection [23,51].

### Home range overlap

Leopards showed substantial home range overlaps in Tandoureh. The estimates of home range overlaps in the current study were considerably larger than reported in many previous studies on leopards (Table 3). There are two possible explanations. The majority of previous studies were based on VHF telemetry, which may miss significant animal movements and consequently result in smaller home range overlap estimates [52]. Likewise, the conventional KDE and MCP estimation generally provide a lower bound for the estimate of home range area [40], and consequently result in overlap reduction. Alternatively, the higher home range overlap observed in Tandoureh is attributable to the topographic features of this rugged landscape that can facilitate co-existence of multiple individuals. Landscape heterogeneity and topographic features can provide restricted detectability for leopards and promote their spatiotemporal overlap.

**Table 3:** A review of comparative intra-male home range overlap at different study sites, based on telemetry techniques. MCP = minimum convex polygon, KDE = Kernel density estimation and AKDE = auto-correlated Kernel density estimation.

Location/country	Tracking method	Estimation method	Intra-male home range overlap (%)	Study
Kruger National Park	VHF telemetry	MCP	21.5	[26]
Lolldaiga Hills, Kenya	VHF telemetry	MCP, grid-cell and harmonic mean methods	0–9	[27]
Kaeng Krachan National Park, Thailand	VHF telemetry	MCP	40	[53]
Waterberg Plateau, Namibia	VHF telemetry	MCP	24 ± 13	[31]
Bardia National Park, Nepal	VHF telemetry	KDE	7	[47]
Phinda Game Reserve, South Africa	VHF & GPS telemetry	KDE	4.5 ± 1.5	[30]
Tandoureh National Park, Iran	GPS telemetry	AKDE	43.3 ± 5.9	This study

Predation occurred mostly in parts of the home range used exclusively by each leopard, i.e. home range core areas. We know of only two other studies evaluating the spatial configuration of hunting grounds in regard to felids’ core areas. Predation events were reported to be more frequent outside core areas for both other case studies, i.e. jaguar *Panthera onca* [54] and puma *Puma concolor* [55]. Amongst home ranges with high degrees of spatial overlap, exclusive hunting areas can facilitate coexistence of multiple individuals. Competition over resources, including kills, is a cause of intraspecific agonistic behavior in leopards [56] and resulted in the death of the only collared female leopard in Tandoureh.

An obvious limitation of our study is the small sample size of GPS collared leopards, of which most were male (five out of six). Clearly the findings on a single female risk influence by individual idiosyncrasy [57]. Nevertheless, besides Simcharoen et al. [46] pioneering work (with eight collared leopards), our study is the most intensive study ever conducted on Asian leopards in terms of sample size and collaring period, reflecting the difficulty of working in the harsh landscapes in which Asian leopards persist.

### Conclusion and synthesis

Home range, as described by Powell and Mitchell [58], is “that part of an animal’s cognitive map of its environment that it chooses to keep updated”. We speculate that our findings support an ‘anchoring’ and ‘adjustment’ paradigm in the use of space. Anchoring and adjustment, are cognitive biases in the assessment of risk first described in humans by Tversky and Kahneman [59]. According to this psychological heuristic, when people assess the magnitude of a risk, they start with an implicitly suggested reference point (the "anchor") and make adjustments to it to reach their estimate.

We do not imply the same mechanisms underlying this heuristic in humans apply to leopards, not least as the concept of ‘rational choice’ has a different meaning in non-humans [60]. If only in the form of a helpful analogy, the national park may be functioning as an ‘anchor’ for leopards (and probably many other animals) while they adjust their cognitive space use beyond the park boundaries (where, incidentally, they are not often associated with stock raiding). None of our collared leopards was killed by humans, whereas in the absence of properly managed protected areas, leopards can experience high rates of human-induced mortality in multi-use lands [8,61].

Such anchoring and adjustment behavior supports the proposition that, in Asia’s rugged landscapes, a combination of land sparing and land sharing strategies at multiple spatiotemporal scales has the potential to ensure viability of leopards and other big cats. Properly-managed conservation areas (spared lands) are of paramount importance for securing high densities of large carnivores, insofar as they control poaching of carnivores and their prey species. Nonetheless, their space use outside-conservation areas must be managed through promoting the existence of carnivores in human-dominated landscapes (“land sharing”), with minimized levels of conflict with stock breeders.

With 25% of the global land surface area, mountain ecosystems support a wide range of ecosystem services and biodiversity [62]. Climate change is expected to have a radical effect on biodiversity in mountainous areas [63], forcing northward and upward range shifts in many mammalian species [64–66], including humans [67]. Asian mountains can serve as climate refugia for big cats [68,69], despite the fact that only one third of their current extant range remains as suitable habitat in the next half century [69,70]. Land use change is the main driving factor for range losses in threatened mammalian carnivores [71]. Conservation policy should clearly be proactive wherever possible for sparing montane refugia, preferably larger and better-connected areas, to anchor a high density of breeding nuclei of large cats in Asia’s rugged landscapes. Nonetheless, many montane protected areas are not large enough to meet extensive spatial requirements, high energy needs and hierarchical social interaction of big cats [2]. Therefore, bolstering the coexistence model (i.e. land sharing) is inevitable in order to support viability of both big cats and human communities, which are strongly dependent on reduced water resources in high altitudes. Future research might usefully explore the interaction between the land sharing and sparing, and how it can support both larger carnivore viability and human livelihoods, particularly in the context of montane landscapes.

## References

[1] Balme GA, Slotow R, Hunter LTB. Edge effects and the impact of non-protected areas in carnivore conservation: leopards in the Phinda? Mkhuze Complex, South Africa. Anim Conserv. 2010;13: 315–323.

[2] Johansson Ö, Rauset GR, Samelius G, McCarthy T, Andrén H, Tumursukh L, et al Land sharing is essential for snow leopard conservation. Biol Conserv. 2016;203: 1–7.

[3] Loveridge AJ, Valeix M, Davidson Z, Murindagomo F, Fritz H, Mac Donald DW. Changes in home range size of African lions in relation to pride size and prey biomass in a semi-arid savanna. Ecography. 2009 32: 953–962.

[4] Nilsen EB, Herfindal I, Linnell JDC. Can intra-specific variation in carnivore home-range size be explained using remote-sensing estimates on environmental productivity. Ecoscience. 2005;12: 68–75.

[5] Benson JF, Chamberlain MJ, Leopold BD. Regulation of space use in a solitary felid: population density or prey availability. Anim Behav. 2006;71: 685–693.

[6] Goodrich J, Miquelle D, Smirnov E, Kerley L, Quigley H, Hornocker MG. Spatial structure of Amur (Siberian) tigers (Panthera tigris altaica) on Sikhote-Alin Biosphere Zapovednik, Russia. J Mammal. 2010;91: 737–748.

[7] Allen AM, Månsson J, Sand H, Malmsten J, Ericsson G, Singh NJ. Scaling up movements: from individual space use to population patterns. Ecosphere. 2016;7.

[8] Swanepoel LH, Somers MJ, van Hoven W, Schiess-Meier M, Owen C, Snyman A, et al Survival rates and causes of mortality of leopards Panthera pardus in southern Africa. Oryx. 2015;49: 595–603.

[9] Baskin L, Danell K. Ecology of ungulates: a handbook of species in Eastern Europe and Northern and Central Asia. Springer Science & Business Media; 2003.

[10] Mallon DP, Zhigang J. Grazers on the plains: challenges and prospects for large herbivores in Central Asia. J Appl Ecol. 2009;46: 516–519.

[11] Chapron G, Kaczensky P, Linnell JDC, Von Arx M, Huber D, Andrén H, et al Recovery of large carnivores in Europe’s modern human-dominated landscapes. Science. 2014 346: 1517–1519. doi: 10.1126/science.1257553 25525247 10.1126/science.1257553

[12] Gompper ME, Belant JL, Kays R. Carnivore coexistence: America’s recovery. Science. 2015 347: 382–383. 10.1126/science.347.6220.382-b 25613881

[13] Phalan B, Onial M, Balmford A, Green RE. Reconciling food production and biodiversity conservation: land sharing and land sparing compared. Science. 2011 333: 1289–1291. doi: 10.1126/science.1208742 21885781 10.1126/science.1208742

[14] Packer C, Loveridge A, Canney S, Caro T, Garnett ST, Pfeifer M, et al Conserving large carnivores: dollars and fence. Ecol Lett. 2013;16: 635–641. doi: 10.1111/ele.12091 23461543 10.1111/ele.12091

[15] Durant SM, Becker MS, Creel S, Bashir S, Dickman AJ, Beudels‐Jamar RC, et al Developing fencing policies for dryland ecosystems. J Appl Ecol. 2015;52: 544–551.

[16] McCarthy T, Mallon D, Jackson R, Zahler P, McCarthy K. Panthera uncia. In: The IUCN Red List of Threatened Species 2017: e.T22732A50664030 [Internet]. 2017 [cited 9 Feb 2018]. Available: http://dx.doi.org/10.2305/IUCN.UK.2017-2.RLTS.T22732A50664030.en

[17] Jacobson AP, Gerngross P, Lemeris JR, Schoonover RF, Anco C, Breitenmoser-Würsten C, et al Leopard (Panthera pardus) status, distribution, and the research efforts across its range. PeerJ. 2016;2016: e1974 doi: 10.7717/peerj.1974 10.7717/peerj.1974 PMC4861552 27168983

[18] Hamidi AK, Ghoddousi A, Soufi M, Ghadirian T, Jowkar H, Ashayeri S. Camera trap study of Persian leopard in Golestan National Park, Iran. Cat News. 2014;60: 12–14.

[19] Alexander JS, Gopalaswamy AM, Shi K, Riordan P. Face Value: Towards Robust Estimates of Snow Leopard Densities. PLoS One. 2015;10: e0134815 doi: 10.1371/journal.pone.0134815 26322682 10.1371/journal.pone.0134815 PMC4554729

[20] Karanth KU, Nichols JD, Kumar SN, Link WA, Hines JE. Tigers and their prey: Predicting carnivore densities from prey abundance. PNAS. 2004;101: 4854–4858. doi: 10.1073/pnas.0306210101 15041746 10.1073/pnas.0306210101 PMC387338

[21] Tobler MW, Powell GVN. Estimating jaguar densities with camera traps: Problems with current designs and recommendations for future studies. Biol Conserv. 2013;159: 109–118.

[22] Jiang G, Qi J, Wang G, Shi Q, Darman Y, Hebblewhite M, et al New hope for the survival of the Amur leopard in China. Sci Rep. 2015;5: 15475 doi: 10.1038/srep15475 26638877 10.1038/srep15475 PMC4670984

[23] Farhadinia M, Ahmadi M, Sharbafi E, Khosravi S, Alinezhad H, Macdonald D. Leveraging trans-boundary conservation partnerships: Persistence of Persian leopard (Panthera pardus saxicolor) in the Iranian Caucasus. Biol Conserv. 2015;191: 770–778.

[24] Rostro-García S, Kamler JF, Ash E, Clements GR, Gibson L, Lynam AJ, et al Endangered leopards: range collapse of the Indochinese leopard (Panthera pardus delacouri) in Southeast Asia. Biol Conserv. 2016;201: 293–300.

[25] Stein AB, Hayssen V. Panthera pardus (Carnivora: Felidae). Mamm Species. 2013;45: 30–48.

[26] Bailey TN. The African leopard: ecology and behavior of a solitary felid Columbia University Press; 1993.

[27] Mizutani F, Jewell PA. Home range and movements of leopards (Panthera pardus) on a livestock ranch in Kenya. J Zool. 1998;244: 269–286.

[28] Rozhnov V V, Chistopolova MD, Lukarevskii VS, Hernandez-Blanco JA, Naidenko S V, Sorokin PA. Home range structure and space use of a female Amur leopard, Panthera pardus orientalis (Carnivora, Felidae). Biology Bulletin. 2015 42: 821–830.

[29] Balme GA, Slotow R, Hunter LTB. Impact of conservation interventions on the dynamics and persistence of a persecuted leopard (Panthera pardus) population. Biol Conserv. 2009;142: 2681–2690.

[30] Fattebert J, Balme GA, Robinson HS, Dickerson T, Slotow R, Hunter LTB. Population recovery highlights spatial organization dynamics in adult leopards. J Zool. 2016;299: 153–162.

[31] Marker LL, Dickman AJ. Factors affecting leopard (Panthera pardus) spatial ecology, with particular reference to Namibian farmlands. South African J Wildl Res. 2005;35: 105–115.

[32] Frank L, Simpson D, Woodroffe R. Foot snares: an effective method for capturing African lions. Wildl Soc Bull. 2003;31: 309–314.

[33] Farhadinia M, Memarian I, Hobeali K, Shahrdari A, Ekrami B, Kaandorp J, et al GPS collars reveal transboundary movements by Persian leopards in Iran. Cat News. 2017;65: 28–30.

[34] Stander PE. Field age determination of leopards by tooth wear. Afr J Ecol. 1997;35: 156–161.

[35] Knopff KH, Knopff AA, Warren MB, Boyce MS. Evaluating global positioning system telemetry techniques for estimating cougar predation patterns. J Wildl Manage. 2009;73: 586–597.

[36] Cain III JW, Krausman PR, Jansen BD, Morgart JR. Influence of topography and GPS fix interval on GPS collar performance. Wildl Soc Bull. 2005;33: 926–934.

[37] Bjørneraas KK, Van Moorter B, Rolandsen CM, Herfindal I. Screening Global Positioning System Location Data for Errors Using Animal Movement Characteristics. J Wildl Manage. 2010;74: 1361–1366.

[38] R Development Core Team. R: A language and environment for statistical computing. 2013;

[39] Worton BJ. Kernel methods for estimating the utilization distribution in home-range studies. Ecology. 1989;70: 164–168.

[40] Fleming CH, Fagan WF, Mueller T, Olson KA, Leimgruber P, Calabrese JM. Rigorous home range estimation with movement data: a new autocorrelated kernel density estimator. Ecology. 2015;96: 1182–1188. 26236833 10.1890/14-2010.1

[41] Calabrese JM, Fleming CH, Gurarie E. ctmm: an r package for analyzing animal relocation data as a continuous-time stochastic process. Methods Ecol Evol. 2016;7: 1124–1132.

[42] Fleming CH, Calabrese JM. A new kernel density estimator for accurate home-range and species-range area estimation. Methods Ecol Evol. 2017;8: 571–579.

[43] Vander Wal E, Rodgers AR. An individual-based quantitative approach for delineating core areas of animal space use. Ecol Modell. 2012;224: 48–53.

[44] Fieberg J, Kochanny CO. Quantifying home-range overlap: the importance of the utilization distribution. J Wildl Manage. 2005;69: 1346–1359.

[45] Karanth KU, Sunquist ME. Behavioural correlates of predation by tiger (Panthera tigris), leopard (Panthera pardus) and dhole (Cuon alpinus) in Nagarahole, India. J Zool. 2000;250: 255–265.

[46] Simcharoen S, Barlow ACD, Simcharoen A, Smith JLD. Home range size and daytime habitat selection of leopards in Huai Khaeng Wildlife Sanctuary, Thailand. Biol Conserv. 2008;141: 2242–2250.

[47] Odden M, Wegge P. Spacing and activity patterns of leopards Panthera pardus in the Royal Bardia National Park, Nepal. Wildlife Biology. 200511: 145–152.

[48] Hunter L. Carnivores of the World. Princeton University Press Princeton; 2011.

[49] Kelt DA, Van Vuren DH. The ecology and macroecology of mammalian home range area. Am Nat. 2001;157: 637–645. doi: 10.1086/320621 18707280 10.1086/320621

[50] Farhadinia M, Kaboli M, Karami M, Farahmand H. Patterns of sexual dimorphism in the Persian Leopard (Panthera pardus saxicolor) and implications for sex differentiation. Zool Middle East. 2014;60: 195–207.

[51] Gavashelishvili A, Lukarevskiy V. Modelling the habitat requirements of leopard Panthera pardus in west and central Asia. J Appl Ecol. 2008;45: 579–588.

[52] Kochanny CO, Delgiudice GD, Fieberg J. Comparing global positioning system and very high frequency telemetry home ranges of white-tailed deer. J Wildl Manage. BioOne; 2009;73: 779–787.

[53] Grassman LI. Ecology and behavior of the Indochinese leopard in Kaeng Karchan National Park, Thailand. Nat Hist Bull Siam Soc. 1999;47: 77–93.

[54] de Azevedo FCC, Murray DL. Spatial organization and food habits of jaguars (Panthera onca) in a floodplain forest. Biol Conserv. 2007;137: 391–402.

[55] Pierce BM, Bleich VC, Bowyer RT. Social organization of mountain lions: does a land-tenure system reuglate population size? Ecology. 2000;81: 1533–1543.

[56] Steyn V, Funston PJ. A case of cannibalism in leopards. South African J Wildl Res. 2006;36: 189–190.

[57] Börger L, Franconi N, De Michele G, Gantz A, Meschi F, Manica A, et al Effects of sampling regime on the mean and variance of home range size estimates. J Anim Ecol. 2006;75: 1393–1405. doi: 10.1111/j.1365-2656.2006.01164.x 17032372 10.1111/j.1365-2656.2006.01164.x

[58] Powell RA, Mitchell MS. What is a home range? J Mammal. 2012;93: 948–958.

[59] Tversky A, Kahneman D. Judgment under uncertainty: Heuristics and biases. Science. 1974185: 1124–1131. 10.1126/science.185.4157.1124 17835457

[60] Stanovich KE. Why humans are (sometimes) less rational than other animals: Cognitive complexity and the axioms of rational choice. Think Reason. 2013;19: 1–26.

[61] Williams ST, Williams KS, Lewis BP, Hill RA. Population dynamics and threats to an apex predator outside protected areas: implications for carnivore management. R Soc Open Sci. 2017;4: 161090 doi: 10.1098/rsos.161090 28484625 10.1098/rsos.161090 PMC5414262

[62] Körner C. The use of “altitude” in ecological research. Trends Ecol Evol. 2007;22: 569–574. doi: 10.1016/j.tree.2007.09.006 17988759 10.1016/j.tree.2007.09.006

[63] Thuiller W, Lavorel S, Araújo MB, Sykes MT, Prentice IC. Climate change threats to plant diversity in Europe. Proc Natl Acad Sci U S A. National Acad Sciences; 2005;102: 8245–8250. doi: 10.1073/pnas.0409902102 15919825 10.1073/pnas.0409902102 PMC1140480

[64] Hickling R, Roy DB, Hill JK, Fox R, Thomas CD. The distributions of a wide range of taxonomic groups are expanding polewards. Glob Chang Biol. 2006;12: 450–455.

[65] Marino J, Bennett M, Cossios D, Iriarte A, Lucherini M, Pliscoff P, et al Bioclimatic constraints to Andean cat distribution: a modelling application for rare species. Divers Distrib. 2011;17: 311–322.

[66] Luo Z, Jiang Z, Tang S. Impacts of climate change on distributions and diversity of ungulates on the Tibetan Plateau. Ecol Appl. 2015;25: 24–38. 26255355 10.1890/13-1499.1

[67] Nogués-Bravo D, Araújo MB, Errea MP, Martinez-Rica JP. Exposure of global mountain systems to climate warming during the 21st Century. Glob Environ Chang. 2007;17: 420–428.

[68] Forrest JL, Wikramanyake E, Shrestha R, Areendran G, Gyeltshen K, Maheshwari A, et al Conservation and climate change: Assessing the vulnerability of snow leopard habitat to treeline shift in the Himalaya. Biol Conserv. 2012;150: 129–135.

[69] Li J, McCarthy TM, Wang H, Weckworth B V, Schaller GB, Mishra C, et al Climate refugia of snow leopards in High Asia. Biol Conserv. 2016;203: 188–196.

[70] Ebrahimi A, Farashi A, Rashki A. Habitat suitability of Persian leopard (Panthera pardus saxicolor) in Iran in future. Environ Earth Sci. 2017;76: 697.

[71] Di Minin E, Slotow R, Hunter LTB, Pouzols FM, Toivonen T, Verburg PH, et al Global priorities for national carnivore conservation under land use change. Sci Rep. 2016;6: 23814 doi: 10.1038/srep23814 27034197 10.1038/srep23814 PMC4817124

